# Prospective Cohort study of Predictors of Follow-Up Diagnostic Colonoscopy from a Pragmatic Trial of FIT Screening

**DOI:** 10.1038/s41598-020-59032-0

**Published:** 2020-02-12

**Authors:** Elizabeth A. O’Connor, Carrie M. Nielson, Amanda F. Petrik, Beverly B. Green, Gloria D. Coronado

**Affiliations:** 1 Kaiser Permanente Center for Health Research, 3800 N. Interstate Avenue, Portland, OR 97227 USA; 2 Kaiser Permanente Washington Health Research Institute, 1730 Minor Avenue, Suite 1600, Seattle, WA 98101 USA

**Keywords:** Cancer prevention, Cancer screening

## Abstract

The goal of this study was to explore diagnostic colonoscopy completion in adults with abnormal screening fecal immunochemical test (FIT) results. This was a secondary analysis of the Strategies and Opportunities to Stop Colon Cancer in Priority Populations (Stop CRC) study, a cluster-randomized pragmatic trial to increase uptake of CRC screening in federally qualified community health clinics. Diagnostic colonoscopy completion and reasons for non-completion were ascertained through a manual review of electronic health records, and completion was compared across a wide range of individual patient health and sociodemographic characteristics. Among 2,018 adults with an abnormal FIT result, 1066 (52.8%) completed a follow-up colonoscopy within 12 months. Completion was generally similar across a wide range of participant subpopulations; however, completion was higher for participants who were younger, Hispanic, Spanish-speaking, and had zero or one of the Charlson medical comorbidities, compared to their counterparts. Neighborhood-level predictors were not associated with diagnostic colonoscopy completion. Thus, completion of a diagnostic colonoscopy was relatively low in a large sample of community health clinic adults who had an abnormal screening FIT result. While completion was generally similar across a wide range of characteristics, younger, healthier, Hispanic participants tended to have a higher likelihood of completion.

## Introduction

Colorectal cancer (CRC) incidence and mortality have declined markedly among Americans ages 50 and older since 1990, a victory partially attributable to improved screening rates^1^. However, the burden of CRC has not been alleviated among those in the lowest socioeconomic groups^2,3^. Screening adherence has been demonstrated to reduce the risk of CRC incidence and death, consistent with the assumption that removing pre-cancerous adenomas prevents their progression to carcinoma^4^. And although CRC screening has increased to the point that those with the highest income meet the Healthy People 2020 goal of 70% screen-compliance, fewer than 40% of those who live below the poverty level are adequately screened^5^.

Annual fecal immunochemical testing (FIT) is a cost-effective screening strategy with relatively low patient burden^6^. Therefore, increasing the uptake of FIT testing in primary care and safety-net clinics has been the focus of several studies^7–9^. An essential corollary to FIT uptake is the delivery of timely follow-up diagnostic colonoscopy for individuals with a positive FIT result. This follow-up makes it possible to reduce the risk of colorectal cancer and prevent more advanced-stage disease; low follow-up colonoscopy completion substantially reduces the effectiveness of FIT screening^10,11^. In addition, lengthy intervals (e.g., >10 months) between the FIT positive result and follow-up colonoscopy can increase the risk of CRC or advanced-stage disease^10^. The proportion completing a follow-up colonoscopy after a FIT-positive result has been reported to be approximately 30% to 60% in safety-net and Veterans Affairs health care systems^12–16^, rising to ~80% in integrated health care organizations^17,18^. However, interventions, such as nurse navigators, have achieved up to 91% follow-up colonoscopy completion^17,19^. Clearly, health center systems and resources affect follow-up colonoscopy completion.

In addition to delivery-system-level variation, patient and clinician factors influence follow-up colonoscopy adherence. Patients and clinicians in low-resource clinical settings who comply with FIT screening face additional barriers to completing a timely follow-up diagnostic colonoscopy essential for cancer prevention. Low socioeconomic status (SES) and lack of continuity in care correspond to lower odds of completing follow-up colonoscopy^20,21^. Within safety-net health care settings, follow-up completion also differs by patient factors, such as race/ethnicity, sex, age, and comorbidities^13,14,22^, and system or clinician factors, such as referral and scheduling practices^14^.

In a pragmatic clinical trial of FIT mailing in federally qualified health centers, we have demonstrated increased FIT uptake^9^, with between 8% and 21% of individuals screening positive^23^. Given the importance of follow-up colonoscopy in realizing the benefits of improved FIT screening, we investigated individual and neighborhood factors associated with timely follow-up colonoscopy completion.

## Methods

The Strategies and Opportunities to Stop Colon Cancer in Priority Populations (Stop CRC) study is a 26-clinic pragmatic cluster-randomized study of CRC screening in federally qualified community health centers (CHC) in Oregon and California, USA. Stop CRC was designed to test the use of a direct-mail approach to CRC screening as compared to usual care^24^. The Stop CRC intervention did not include active strategies to increase uptake of follow-up colonoscopies among those with a positive FIT result With few exceptions, Stop CRC clinics used one of three FIT brands: InSure (Enterix, Inc., Edison, NJ; positivity threshold 50 µg hHb/g feces), OC-Micro (Polymedco, Inc., Cortlandt Manor, NY; 20 µg hHb/g), and Hemosure (Hemosure, Inc., Irwindale, CA; 50 µg hHb/g). The current study is a prospective cohort analysis examining completion of follow-up colonoscopy among participants in both the intervention and control groups who had abnormal FIT results.

The Institutional Review Board of Kaiser Permanente Northwest (KPNW IRB) approved all study activities, and participating clinics ceded human subjects review authority to the KPNW IRB. We obtained a waiver of informed consent from the KPNW IRB. Primary outcomes of the Stop CRC trial have been reported^9^.

### Participant eligibility

Within each CHC included in Stop CRC, individuals were eligible if they were (1) 50–74 years old, (2) had attended a clinic visit in the previous year, and (3) were due for CRC screening. Being due for screening was defined as having no evidence in the electronic health record (EHR) of either (1) a fecal test in the previous year, (2) an order for a fecal test in the previous 6 months, (3) a flexible sigmoidoscopy in the previous 4 years, (4) a colonoscopy in the previous 9 years, or (5) an order for a sigmoidoscopy/colonoscopy in the previous year. Individuals were excluded if they had EHR evidence of several health conditions that made them poor candidates for fecal testing (e.g., a history of CRC, colon disease, or renal failure)^25^.

### Analytic sample

The current study is limited to the 2,018 participants who returned a FIT and had an abnormal FIT result between February 4, 2014, and August 3, 2016.

### Data sources

All data were extracted from EHRs, accessed in collaboration with the Oregon Community Health Information Network (OCHIN). OCHIN is a non-profit health center network with an organization wide EHR that allows researchers to access sociodemographic, clinical, and utilization data across all OCHIN clinic sites. Neighborhood variables were obtained from the ADVANCE Clinical Data Research Network^26^, based on the participant’s address on February 4, 2014, or the closest record to that date.

### Outcomes

The primary outcome was a completed colonoscopy within 12 months of the participant’s first positive FIT result. The EHR of every person with an abnormal FIT was manually examined for evidence of a completed colonoscopy. We relied on manual chart abstraction for this outcome rather than electronic clinical databases because colonoscopy services were typically referred to clinics outside the OCHIN network, and in those cases colonoscopy results may have been manually entered into the health record without using the standard procedure code fields that are exported to searchable databases.

In addition, the EHR was examined for notation related to the reason a follow-up colonoscopy was not completed. Reasons were coded into the following categories: the individual declined, had a recent colonoscopy, could not be found and notified, did not come to their scheduled colonoscopy appointment, the clinician considered the individual to be a poor candidate for a colonoscopy, the individual was given a second FIT to confirm the abnormal result before scheduling a colonoscopy, and inadequate or intolerance to prep for the colonoscopy.

### Predictors of service use

#### Individual characteristics

Individual characteristics, including demographics, insurance status, income relative to federal poverty level, and office visits in the year prior to initial eligibility determination, were ascertained through OCHIN EHR. Health conditions in the year prior to eligibility were determined by calculating the Charlson comorbidity index, using the Elixhauser coding algorithms^27^. EHR evidence of screening behaviors — Pap within the previous 3 years, mammogram within the previous 2 years, and flu shot within the previous year — were also collected.

#### Neighborhood characteristics

Most neighborhood characteristics were collected at the census tract level, and included Gini Index (a measure of income inequality)^28^, unemployment rate, population density, median household income, percent of college graduates, and the percentage of residents who are at or below the poverty level. The variable for rate of Emergency Department visits per 1000 Centers for Medicaid and Medicare (CMS) enrollees was collected at the county level. Neighborhood characteristics were defined based on the participant’s address at the time of enrollment, which was linked via geocoding to variables from the American Community Survey census data^29^ and the CMS Geographic Variation database^30^. These neighborhood-level variables were dichotomized based on associated statistics in the United States, as close to the year 2014 as possible for consistency with the timeline of the study (Table 1).

**Table 1:** Description of neighborhood-level moderators and cut-point selected for dichotomizing.

Moderator	Description and cut point for dichotomizing
Gini Inequality	The Gini index, or index of income concentration, is a statistical measure of income inequality ranging from 0 to 1. A measure of 1 indicates perfect inequality, i.e., one household having all the income and rest having none. A measure of 0 indicates perfect equality, i.e., all households having an equal share of income^28^. Dichotomized at 0.4106, the World Bank estimate of Gini for the U.S. in 2013^37^.
Unemployment Rate	Number of unemployed people as a percentage of the civilian labor force. Dichotomized at 6.6, the U.S. seasonally adjusted unemployment rate in January, 2014^38^.
Percent College Grad	Percent with bachelor’s degree or Higher. Dichotomized at 41.0, the percent of U.S. citizens age 55–64 with tertiary education, 2014^39^.
Population Density	Total population divided by the land area measured in square miles. Dichotomized at 1000 people/square mile, the definition of a rural tract.
Median Household Income	50^th^ percentile household income for the census tract. Dichotomized at 68,426, the median household income in the U.S. in 2014, for family households^40^.
Poverty (Percent)	Percent in census tract living in poverty. Census Bureau uses a set of money income thresholds that vary by family size and composition to determine who is in poverty. If the total income for a family or unrelated individual fall below the relevant poverty threshold, then the family (and every individual in it) or unrelated individual is considered in poverty. Dichotomized at 17.6%, a median split in our study sample.
ED Visits per 1,000 Enrollees	Number of emergency department visits per 1000 Medicaid/Medicare enrollees. Dichotomized at 416, the U.S. average in 2013^41^.

### Statistical analysis

Simple frequency tables were constructed to examine the reasons for non-completion of a follow-up colonoscopy and to describe the demographic, socioeconomic, health-related, and neighborhood-level predictors. To examine factors that influenced the likelihood of completing a follow-up colonoscopy, relative risks were calculated using log-linked binomial models, with the dichotomous outcome indicating follow-up colonoscopy completion, and the model included one predictor at a time, controlling for age, sex, and health system. PROC GENMOD in SAS version 9.4 was used for this analysis. Most predictors were dichotomous (e.g., presence or absence of a medical condition) or categorical in nature, and others were dichotomized according to a clinically or otherwise meaningful logic, such as BMI at the standard cut-off for obesity. Predictors that had 20 or fewer participants for either level of the variable were not included in further analyses. All significance testing was 2-tailed, and results were considered statistically significant if the p-value was 0.05 or less.

### Compliance with ethical standards

All procedures performed in studies involving human participants were in accordance with the ethical standards of the institutional and/or national research committee and with the 1964 Helsinki declaration and its later amendments or comparable ethical standards. This article does not contain any studies with animals performed by any of the authors.

## Results

Overall, 2,018 (17.7%) of 11,427 participants who completed a FIT had an abnormal FIT result. Of these, 1,066 (52.8%) completed a follow-up diagnostic colonoscopy within 12 months of their FIT test. The proportion completing a FIT ranged from 47.2% to 60.9% (data not shown) across health systems, highlighting the importance of controlling for health system in subsequent analyses. The median time to completion was 72 days (interquartile range, 49 to 120).

Reasons for noncompletion were found in the EHR of 546 of the 952 participants who did not complete a follow-up colonoscopy (Table 2). The most common reason for non-completion was that the patient declined, noted for 237 (43.4%) of the 546 participants with a reason recorded. Twenty-two percent had a recent colonoscopy, 12.5% could not be found for notification, and 10.3% did not show up to a scheduled appointment. When the participants who declined the follow-up colonoscopy due to self-report of a recent colonoscopy were removed from the denominator, completion increased slightly, to 56.2% (1066/1897).

**Table 2:** Reason for non-completion of colonoscopy, among those with a reason noted in the health record (n = 546).

Reason	No. Participants	%
Patient declined	237	43.4
Recent prior colonoscopy	121	22.2
Unable to be notified	68	12.5
No-show	56	10.3
Patient is a poor candidate	28	5.1
Given second FIT	23	4.2
Inadequate prep or tolerance	13	2.4

Across a wide range of potential predictor categories, follow-up colonoscopy completion fell between 45% and 55% for most participant subgroups. The percent completing a follow-up colonoscopy for demographic and socioeconomic subgroups and the between-group effects are shown in Table 3. Follow-up colonoscopy completion was lower for adults age 65–74 (45.3%) than those aged 50–64 (54.7%, adjusted RR [aRR] = 0.84, 95% CI 0.74 to 0.94), and the highest completion was seen for participants who were Hispanic (63.1%, vs. 52.0% non-Hispanic, aRR = 1.19, 95% CI 1.04 to 1.36) and Spanish-speaking (67.0%, vs. 52.2% and 51.0% for English and other languages, respectively, aRR = 1.30, 95% CI 1.12 to 1.50 relative to English speakers). Participants with a Charlson comorbidity index of 2 or more were less likely to complete a follow-up colonoscopy (47.4%) than those with an index value of zero or one (54.5%, aRR = 0.88, 95% CI 0.79 to 0.98, Table 4). The only specific health condition that was statistically significantly associated completion of a follow-up colonoscopy was renal disease; 39.1% of participants with a diagnosis of renal disease completed a follow-up colonoscopy, compared with 53.4% of those without (aRR = 0.75, 95% CI 0.58 to 0.97). None of the neighborhood-level predictors were associated with completion of a follow-up colonoscopy (Table 5).

**Table 3:** Demographic and socioeconomic predictors of follow-up colonoscopy, among participants with a positive FIT (n = 2018^a^).

	No. with Abnormal FIT	No. (%) colonoscopy documented	aRR^b^	95% CI
**Age**				
50–64	1623	887 (54.7)	1.00	
65–74	395	179 (45.3)	0.84	(0.74–0.94)
**Gender**				
Male	961	552 (53.5)	1.00	
Female	1057	514 (52.2)	1.02	(0.94–1.11)
**Race**				
White	1670	901 (54.0)	1.00	
Asian	184	89 (48.4)	0.94	(0.80–1.11)
African American	125	59 (47.2)	0.87	(0.72–1.06)
Pacific Islander	12	4 (33.3)	0.63	(0.28–1.40)
Native American	27	27 (48.1)	0.93	(0.63–1.38)
**Hispanic**				
No	1869	972 (52.0)	1.0	
Yes	149	94 (63.1)	1.19	(1.04–1.36)
**Language**				
English	1658	866 (52.2)	1.0	
Other	257	131 (51.0)	1.03	(0.90–1.18)
Spanish	103	69 (67.0)	1.30	(1.12–1.50)
**Insurance status**				
Commercial	197	111 (56.3)	1.00	
Medicaid	954	531 (55.7)	1.00	(0.87–1.15)
Medicare	528	250 (47.3)	0.89	(0.76–1.05)
Uninsured	339	174 (51.3)	0.94	(0.80–1.11)
**Federal Poverty Level**				
200%+	230	269 (52.2)	1.00	
100–200%	487	120 (55.2)	1.03	(0.88–1.19)
<100%	879	453 (51.5)	0.96	(0.84–1.11)
Unknown	422	224 (53.1)	0.97	(0.84–1.14)
**Current tobacco use**				
No	1231	655 (53.2)	1.00	
Yes	559	288 (51.5)	0.96	(0.87–1.05)
**Obesity (BMI** > = **30)**				
No	1091	581 (53.3)	1.00	
Yes	927	485 (52.3)	0.97	(0.89–1.05)
**Pap in past 3 years (women** < **65** **yr)**				
No	436	225 (51.6)	1.00	
Yes	394	222 (56.3)	1.07	(0.94–1.21)
**Mammogram in past 2 years (women)**				
No	561	275 (49.0)	1.00	
Yes	496	277 (55.8)	1.12	(1.00–1.26)
**Flu shot in past year**				
No	1274	654 (51.3)	1.00	
Yes	744	412 (55.4)	1.08	(1.00–1.18)
**Number of office visits in past year**				
1–2	821	435 (53.0)	1.00	
3–5	560	292 (52.1)	0.99	(0.89–1.09)
6+	637	339 (53.2)	1.01	(0.91–1.11)

^a^Number of participants is less the 2,018 for some predictors due to missing data

^b^Model adjusted for age, sex, and health system.

**Table 4:** Medical conditions as predictors of follow-up colonoscopy, among participants with a positive FIT.

	No. with Abnormal FIT	% colonoscopy documented	RR	95% CI
**Charlson Index 2**+ (**vs 0–1)**				
No	1535	837 (54.5)	1.00	
Yes	483	229 (47.4)	0.88	(0.79–0.98)
**Congestive heart disease**				
No	1940	1031 (53.1)	1.00	
Yes	78	35 (44.9)	0.85	(0.66–1.08)
**Cerebrovascular disease**				
No	1903	1007 (52.9)	1.00	
Yes	115	59 (51.3)	0.98	(0.82–1.18)
**Peripheral vascular disorder**				
No	1965	1043 (53.1)	1.00	
Yes	53	23 (43.4)	0.88	(0.64–1.20)
**Myocardial Infarction**				
No	1963	1044 (53.2)	1.00	
Yes	55	22 (40.0)	0.74	(0.53–1.02)
**Diabetes**				
No	1494	787 (52.7)	1.00	
Yes	524	279 (53.2)	1.02	(0.93–1.12)
**Diabetes with chronic complications**				
No	1927	1026 (53.2)	1.00	
Yes	91	40 (44.0)	0.82	(0.65–1.04)
**Chronic pulmonary disease (includes asthma, COPD)**				
No	1526	821 (53.8)	1.00	
Yes	492	245 (49.8)	0.93	(0.84–1.03)
**Malignancy, including leukemia and lymphoma**				
No	1941	1027 (52.9)	1.00	
Yes	77	39 (50.6)	0.96	(0.77–1.20)
**Mild liver disease**				
No	1980	1048 (52.9)	1.00	
Yes	38	18 (47.4)	0.87	(0.62–1.22)
**Peptic ulcer disease**				
No	1981	1044 (52.7)	1.00	
Yes	37	22 (59.5)	1.11	(0.85–1.44)
**Renal Disease**				
No	1926	1030 (53.5)	1.00	
Yes	92	36 (39.1)	0.75	(0.58–0.97)
**Rheumatologic disease**				
No	1992	1055 (53.0)	1.00	
Yes	26	11 (42.3)	0.79	(0.50–1.23)
**Depression**				
No	1374	736 (53.6)	1.00	
Yes	644	330 (51.2)	0.94	(0.86–1.03)
**Psychosis**				
No	1953	1028 (52.6)	1.00	
Yes	65	38 (58.5)	1.11	(0.90–1.36)
**Alcohol use disorder**				
No	1988	1047 (52.7)	1.00	
Yes	30	19 (63.3)	1.23	(0.94–1.61)
**Drug use disorder**				
No	1824	972 (53.3)	1.00	
Yes	194	94 (48.5)	0.88	(0.75–1.02)
**Substance Abuse**				
No	1797	956 (53.2)	1.00	
Yes	221	110 (49.8)	0.91	(0.79–1.04)

Note: Model adjusted for age, sex, and health system.

**Table 5:** Neighborhood-level predictors of follow-up colonoscopy, among participants with an abnormal FIT.

	No. with Abnormal FIT	% colonoscopy documented	RR	95% CI
**Gini**				
Less than US median (0.47)	1648	870 (52.8)	1.00	
Greater than US median	321	174 (54.2)	1.02	(0.91–1.13)
**Unemployment rate**				
Less than US median (8.78%)	754	400 (53.1)	1.00	
Greater than US median	1215	644 (53.0)	1.02	(0.94–1.11)
**College degree attainment**				
Less than US median (41%)	1561	826 (52.9)	1.00	
Greater than US median	408	218 (53.4)	0.98	(0.88–1.08)
**Population Density**				
Less than 1,000	483	250 (51.8)	1.00	
Greater than 1,000	1486	794 (53.4)	1.02	(0.91–1.14)
**Medium Home Income**				
Less than US median ($54,102)	1468	782 (53.3)	1.00	
Greater than median	501	262 (52.3)	0.93	(0.84–1.03)
**Percent below federal poverty level**				
Less than US median (18.12%)	888	473 (53.3)	1.00	
Greater than median	1081	571 (52.8)	1.01	(0.93–1.09)
**ED Visits per 1,000 Enrollees**				
Less than US median (621)	1497	813 (54.3)	1.00	
Greater than median	394	198 (50.3)	0.94	(0.76–1.16)

## Discussion

In this pragmatic trial of FIT screening, 52.8% of participants who had an abnormal FIT completed a follow-up colonoscopy within one year. This finding is consistent with other studies in safety-net health clinics, which have found 52% to 58% of individuals completing follow-up colonoscopies at sites in Chicago^22^, Texas^14^, and San Francisco^13^. Follow-up colonoscopy completion is alarmingly low; given the positive predictive value of the FIT tests used in this study for identifying advanced adenoma or cancer (range 0.17 to 0.30^23^), an expected 161 to 286 individuals in the Stop CRC cohort of 11,427 participants are at unnecessary risk of disease progression and cancer mortality.

Completion was generally consistent across a wide range of potential sociodemographic, health, and neighborhood-level predictors, including income and insurance status, which may be hypothesized to have an important influence on completion among this economically disadvantaged population. Hispanic and Spanish-speaking participants had higher than average completion of follow-up colonoscopy, however. Interestingly, in a pilot study of one health center in the Stop CRC study examining colonoscopy completion among individuals with an abnormal FIT (n = 56 with an abnormal FIT), we reported that Hispanics and women had markedly lower odds of completing follow-up colonoscopy within 18 months than non-Hispanic whites and men^31^. In that analysis of a clinic predominantly serving Hispanic individuals, 45% of Hispanics and 70% of non-Hispanic those with a positive FIT received follow-up colonoscopy. It is unclear why results differed so markedly between the pilot and full study, but the fact that colonoscopy completion varied substantially between the pilot and full study hints at the possibility of between-clinic variability in patterns of follow-up colonoscopy. In contrast, another trial found essentially no ethnic difference (70% among Hispanics and 68% among non-Hispanic whites) in the geographically diverse clinics participating in the Population-Based Research Optimizing Screening through Personalized Regimens (PROSPR) consortium^32^. Interestingly, one other study did report an increased likelihood of follow-up colonoscopy among Spanish-speaking individuals in community health centers in Chicago (RR = 1.19, 95% CI 1.04 to 1.36, compared with English-speaking individuals)^22^.

We found a lower likelihood of follow-up colonoscopy among older (age 65 or more) adults. Other studies have also found similar associations^13,14,33^, among adults in the age range covered by the USPSTF screening recommendation. For example, a study of a PROSPR consortium CHC clinics in Texas (n = 1,267) found an OR of 1.59 (95% CI 1.15 to 2.17) comparing those aged 61–64 vs. 50–55^14^. However, other studies have found no age differences in follow-up colonoscopy completion^12,18,34^.

We also found a lower likelihood of follow-up colonoscopy completion among those with a Charlson index of two or more and in those with renal disease. Similarly, in several other studies individuals who lacked follow-up colonoscopy had more comorbidities^14,20^. For example, individuals in the PROSPR consortium clinics with a Charlson comorbidity index score ≥3 were less likely to complete a colonoscopy than those with a score of 0 (HR = 0.70)^35^.

Given the overall low completion of follow-up colonoscopy, effective strategies to increase the likelihood of completion are needed. Several intervention efforts have focused on improving clinician and system factors affecting follow-up colonoscopy referral and scheduling. A recent systematic review found improved colonoscopy completion with the use of personalized letters signed by the participant’s primary care clinician, notification of results by phone, phone reminders to complete a colonoscopy, and the use of patient navigators^36^. Provider-level interventions that sent reminders or performance data to clinicians were also generally associated with increased rates of follow-up colonoscopy completion^36^. In addition, the same review identified several system-level intervention studies that demonstrated increased completion rates over usual care, including an automated referral to a gastroenterologist, replacing a pre-colonoscopy visit with a phone call, creating a registry to track individuals with an abnormal FIT, and multi-component quality improvement interventions. These and other strategies should be more widely implemented, and may be particularly important for older adults and those with multiple medical conditions, who had lower completion rates in this study when there were no specific activities to encourage the completion of follow-up colonoscopies.

### Strengths and limitations

This analysis has several important limitations. This was an exploratory study which examined the impact of many predictors, making it likely that some statistically significant findings were chance occurrences. However, many of our findings replicated those found previously (e.g. age, presence of multiple comorbidities), improving our confidence in the stability of these findings. Nevertheless, the prior evidence base for some predictors are mixed (e.g., Hispanic individuals) and some predictors lacked prior support (e.g., patients with renal disease). Another limitation is that reasons for non-completion was often missing or was non-specific (e.g., “patient declined”). Colonoscopies were typically referred to external clinicians, so getting results and reasons for non-completion into the participant’s EHR required the successful interface across health entities, leaving room for error. The fact that 121 individuals reported that they had a recent colonoscopy, among those with reasons for non-completion, suggests that some colonoscopy reports were not returned to the individual’s primary medical record. Another limitation was the small numbers of patients with some of the health conditions of interest. Frequently, there were fewer than 100 participants with a target health condition, limiting our confidence in these results and the power to detect differences. A fourth limitation is that our sample had very limited racial/ethnic diversity, with 83% identifying as white and 93% as non-Hispanic. The small absolute numbers of non-white and Hispanic participants reduce our confidence in the generalizability of those results to other CHC settings. In addition, the fact that most participants were from low socioeconomic group limits generalizability to more economically advantaged populations.

Finally, another limitation to our study is that there were a large number of site that conducted colonoscopies, but we did not gather information on colonoscopy site such as location or standard procedures (e.g., requirements for pre-visits, escorts, and transportation) that may affect follow-up colonoscopy completion rates. Colonoscopies were typically referred to specialists outside of the primary care clinic. Chart review was conducted at the primary care clinic, and the findings were limited to procedure reports, pathology reports and physician notes. However, we attempted to mitigate both the impact of the variability in how the colonoscopy sites interacted with the health systems and differences in procedures in the health systems by controlling for health system in our analysis.

A strength of our study was that we conducted a manual health record review, which looked beyond procedure or diagnosis codes in standard fields to include images, scanned documents, and other information noted in free text fields. Capture of outcomes was further enhanced by the nature of the OCHIN consortium, which ensures that patients have a single OCHIN medical record that captures utilization and medical information from any OCHIN-affiliated health system. In addition, the richness of the socioeconomic data is a strength of this study. In addition to the geocoded-neighborhood level data, the OCHIN clinics routinely collect individual-level income data, when is then categorized in terms of the federal poverty level.

## Conclusions

In this study set in 26 federally qualified CHCs, only 52.8% of individuals with an abnormal FIT result completed a follow-up colonoscopy within 12 months of the FIT result. The likelihood of completion was generally consistent across a wide range of potential predictors; however, Hispanic and Spanish-speaking individuals showed higher completion, while lower completion was found for those who were older than 65 years, had two or more medical conditions, or had renal disease. Patient refusal was the most commonly cited reason for non-completion. CHCs should consider implementing strategies to increase completion of follow-up colonoscopies after a positive FIT, particularly in older adults and those with multiple existing medical conditions.
